# Deep Reinforcement Learning for UAV Trajectory Design Considering Mobile Ground Users

**DOI:** 10.3390/s21248239

**Published:** 2021-12-09

**Authors:** Wonseok Lee, Young Jeon, Taejoon Kim, Young-Il Kim

**Affiliations:** 1 School of Information and Communication Engineering, Chungbuk National University, Chungju 28644, Korea; dldnjstjr0224@chungbuk.ac.kr (W.L.); jeony9672@chungbuk.ac.kr (Y.J.); ktjcc@chungbuk.ac.kr (T.K.); 2 Electronics and Telecommunications Research Institute, Daejeon 34129, Korea

**Keywords:** unmanned aerial vehicles, reinforcement learning, trajectory optimization

## Abstract

A network composed of unmanned aerial vehicles (UAVs), serving as base stations (UAV-BS network), is emerging as a promising component in next-generation communication systems. In the UAV-BS network, the optimal positioning of a UAV-BS is an essential requirement to establish line-of-sight (LoS) links for ground users. A novel deep Q-network (DQN)-based learning model enabling the optimal deployment of a UAV-BS is proposed. Moreover, without re-learning of the model and the acquisition of the path information of ground users, the proposed model presents the optimal UAV-BS trajectory while ground users move. Specifically, the proposed model optimizes the trajectory of a UAV-BS by maximizing the mean opinion score (MOS) for ground users who move to various paths. Furthermore, the proposed model is highly practical because, instead of the locations of individual mobile users, an average channel power gain is used as an input parameter. The accuracy of the proposed model is validated by comparing the results of the model with those of a mathematical optimization solver.

## 1. Introduction

Due to the advantages of high mobility and easy deployment, unmanned aerial vehicles (UAVs) are emerging as a major component in various applications such as a mobile access point for military operations, a remote structural safety diagnosis, a quick deployment of communication infrastructure for disaster relief, and agricultural monitoring, etc. Naturally, related research works are continually conducted for the efficient utilization of them. In a mobile network, UAV is considered to play important roles as an aerial user and an aerial base station (BS). As an aerial user, UAV is being adopted in various fields such as Structural Health Monitoring (SHM) [1], disaster relief networks [2], agricultural applications [3], search and rescue (SAR) [4], and aerial ad-hoc networks [5,6], etc. Moreover, a network with UAVs, which serve as aerial BSs (UAV-BSs), is becoming a key component in next-generation mobile communication systems. With the ever-demanding request for high-speed mobile communication, next-generation mobile communication technologies are focusing on the efficient use of wide bandwidth. Accordingly, in addition to installing a larger number of BSs, it is necessary to increase the number of line-of-sight (LoS) links between BSs and mobile users. Since a UAV-BS can be located at a high altitude, it has an advantage in supporting LoS links. Compared to a terrestrial BS, a UAV-BS has few installation restrictions and a low installation cost. Furthermore, a UAV-BS can be readily moved to the vicinity of hotspot areas. Considering these features, a network with UAV-BSs is a promising technology for the next-generation networks.

When a UAV acts as a BS, locating the UAV to a proper position is a critical issue. The location of a UAV-BS in a network largely determines the energy consumption of the UAV-BS, the number of users who can be serviced, and the quality-of-experience (QoE) of the users. Accordingly, a lot of research has been conducted to find the optimal position of a UAV-BS, and the main objective of this research is reducing the energy consumption of UAV-BS, improving service quality for users, and maximizing the coverage of UAV-BS, etc. [7,8,9,10,11,12,13,14,15,16]. In [7,8,9,10], optimization algorithms and mathematical optimization solvers are adopted to obtain the optimal location of a UAV-BS. However, the approach of calculating the optimal position of a UAV-BS through an optimization solver has a considerable computational complexity, because whenever the topology of a network changes, it demands re-calculation. On the other hand, reinforcement learning is very efficient in deriving the optimal UAV-BS location and provides a versatile model applicable to various user distributions [17]. A Q-learning algorithm [18] is one of the popular methods for reinforcement. However, Q-learning has a drawback in that the number of states increases explosively as the number of input variables increases, and its memory usage also increases sharply since it should store all the state-action relations in a table. Accordingly, many related works [11,12,13,14,15,16] adopt Deep Q-Network (DQN) [19], which combines Q-learning with an artificial neural network [20].

In [21,22,23], the authors derive the optimal trajectory and path of a UAV-BS using Q-learning. The objectives are to maximize the sum-rate [21], to maximize the QoE of users [22], and to maximize the number and fairness of users served [23]. In these papers, the altitude of a UAV-BS is fixed, and the results are 2-D trajectories of the UAV-BS. By contrast, in this paper, a DQN model producing 3-D trajectories is proposed where the altitude of a UAV-BS is adjusted according to the density of ground users (GUs). In [11,12,13], the optimal UAV-BS deployment algorithms through DQN are proposed. In [11], a network utility and a tolerable convergence speed are maximized. In [12,13], the number of served aerial nodes and an average user throughput is considered. However, these research works do not take the mobility of users into account. Meanwhile, in [14,15,16], the mobility of users is considered in the optimal trajectory design of a UAV-BS. In [14], an uplink sum-rate is maximized by taking both aerial users and GUs into account. In [15], the QoE of aerial users is maximized. However, in [14,15] the location information of all the users and the UAV-BS is required as input parameters for the proposed DQN learning models, and the results do not contain explicit UAV-BS trajectories following mobile users. In [16], an uplink sum-rate is maximized using signal strength as an input parameter for a DQN, and the trajectory of a UAV-BS is presented. However, a simple user mobility model is considered, where GUs move to a specific position only once, and trajectory results for various paths are insufficient. By contrast, the output trajectory of the proposed DQN model in this paper dynamically follows mobile GUs, which move various courses. Moreover, the results of these papers do not show a clear 3-D trajectory of the UAV according to various movement paths of GUs.

In this paper, a UAV-BS trajectory design algorithm, which maximizes QoE considering mobile GUs, is proposed. The contribution of this work is summarized as follows:

- The proposed DQN model exploits an average channel power gain information rather than individual GU position information, which greatly reduces the size of input parameter and computational complexity.
- Reflecting the density of GUs, the adjustment of UAV-BS altitude is enabled. This leads to 3-D trajectory design according to diverse moving patterns of GUs.
- The proposed DQN model learns from a static GUs distribution, then the derived model can be applied to mobile GU scenarios in which the proposed model requires neither the user mobility information nor re-learning for the moving GUs.
- The accuracy of the proposed model is validated by comparing the result of the proposed model with a mathematical optimization solver [24].

Note that applying the proposed DQN model, which is trained in a static GUs distribution, to mobile GU scenarios itself is a great advantage. Because a training a DQN model in a static GU distribution is much easier than training in a mobile GU distribution. In the training of a DQN model with a mobile GUs distribution, the moving pattern of the GUs can be very diverse and the optimal UAV-BS position should be updated in a real-time reflecting the moving GUs.

## 2. System Model

A single UAV-BS denoted as K and a number of N GUs are considered. It is assumed that the UAV-BS communicates with the GUs using time slots of equal length. It is also assumed that the locations of the UAV-BS and the GUs do not change during the time slot duration. Accordingly, 3-D coordinates of the UAV-BS K at time t is $\left(x_{K}\left(t\right),\;y_{K}\left(t\right),\;h_{K}\left(t\right)\right)$, and the GUs are assumed to be on the ground with zero height. The coordinates of the GUs are $\left(x_{i}\left(t\right),\;y_{i}\left(t\right),\;0\right),\;i=1,\cdots ,N$. The distance between the UAV-BS and GU i at time t is expressed as $d_{i}\left(t\right)=\sqrt{{\left(x_{K}\left(t\right)-x_{i}\left(t\right)\right)}^{2}+{\left(y_{K}\left(t\right)-y_{i}\left(t\right)\right)}^{2}+h_{K}^{2}\left(t\right)},\;i=1,\;\cdots ,\;N\;$. For the sake of clarity, the notations and the associated descriptions are provided in Table 1.

### 2.1. Air to Ground Model

The air to ground model considers both LoS and non-LoS (NLoS) characteristics, and the probabilities of connecting LoS and NLoS links are as follows:

(1) $$P_{LOS}\left(\theta_{i}\right)=\frac{1}{1+a\exp\left(-b\left[\theta_{i}-a\right]\right)}\;$$

(2) $$P_{NLOS}\left(\theta_{i}\right)=1-P_{LOS}\left(\theta_{i}\right),$$

where $\theta_{i}$ is an elevation angle between the UAV-BS and GU i, and a and b are constants to be determined according to the surrounding environment (urban, sub-urban, rural, …). It is assumed that the bandwidth and the transmission power of the UAV-BS are equally allocated to all the GUs. Hence, the bandwidth $B_{i}=B/N$ and the transmission power $p_{i}=P/N$ are allocated to GU i, where B and P denote the total bandwidth and the total transmission power, respectively. Then, the received SNR $\Gamma_{i}\left(t\right)$ and the transmission rate $T_{i}\left(t\right)$ of the GU i at time t are expressed as follows:

(3) $$\Gamma_{i}\left(t\right)=\frac{p_{i}g_{i}\left(t\right)}{B_{i}N_{0}},$$

(4) $$T_{i}\left(t\right)=B_{i}\log_{2}\left(1+\Gamma_{i}\left(t\right)\right),$$

where $N_{0}$ is the noise power spectral density and $g_{i}\left(t\right)$ is the channel gain between the UAV-BS and GU i, which is given by [15]:

(5) $$g_{i}\left(t\right)=K_{0}^{-1}d_{i}^{-\alpha }\left(t\right){\left[P_{LOS}\mu_{LOS}+P_{NLOS}\mu_{NLOS}\right]}^{-1},$$

where $K_{0}={\left(\frac{4\pi f_{c}}{c}\right)}^{2}$, α is a path loss exponent, and $\mu_{Los}$ and $\mu_{NLos}$ are attenuation factors for LoS and NLoS, respectively. 

The UAV-BS receives feedback information of the channel power gain from each GU. These received gains are averaged to an average received channel power gain. Therefore, it is possible to simplify the model by reducing the dimension of the input parameter.

### 2.2. QoE Model

QoE is the quality of service experienced by GUs, and mean opinion score (MOS) is a representative metric for QoE. We adopt the MOS model applicable in the TCP protocol proposed in [25]. The simplified MOS model for GU i is as follows [15]:

(6) $${\mathrm{MOS}}_{i}\left(t\right)\;=\;-C_{1}\ln\left[d\left(T_{i}\left(t\right)\right)\right]+C_{2}$$

where $C_{1},\;C_{2}$ are given constants, $d\left(T_{i}\left(t\right)\right)$ is a delay related to the transmission rate for GU i, which is expressed as [26]

(7) $$d\left(T_{i}\left(t\right)\right)=TL/\left(T_{i}\left(t\right)\right)\;$$

where TL is traffic load. 

### 2.3. User Mobility Model

**Figure 1:**
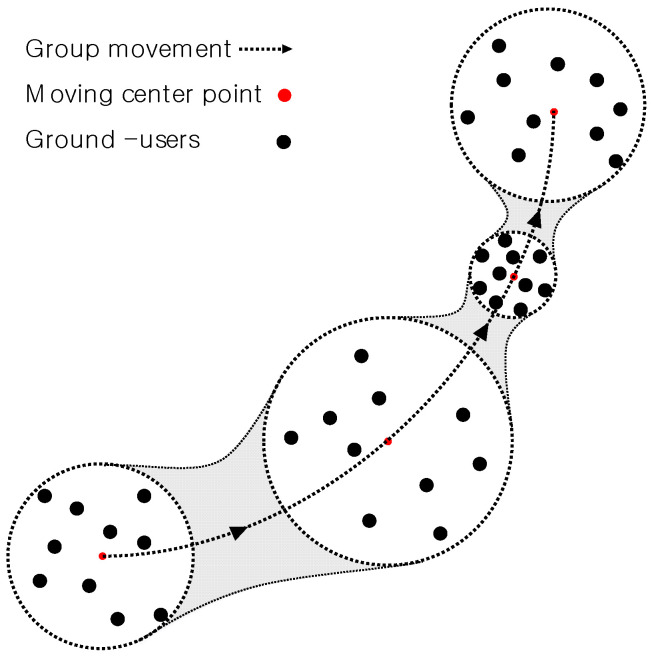
User mobility model with varying user density and random movement around moving center point.

It is assumed that the GUs move randomly within a certain radius around a moving center point while this moving center point moves along a predefined path. The radius may vary over time, and the change of the radius results in the variation of the density of the GUs. The GUs are uniform randomly distributed within the radius. Figure 1 shows the user mobility model schematically.

## 3. Proposed Algorithm

The proposed trajectory design model optimizes not only the horizontal coordinates of the UAV-BS but the altitude of it as well. The model is learned through a DQN by maximizing the MOS of the GUs.

### 3.1. Problem Formulation

In this paper, the goal of the algorithm is to maximize QoE by considering mobile GUs. Hence, the problem formulation maximizes the MOS and can be expressed as:

(8) $$\begin{array}{c} \underset{x_{K}\left(t\right),y_{K}\left(t\right),h_{K}\left(t\right)}{\max}\overset{N}{\underset{i=1}{\sum }}{\mathrm{MOS}}_{i}\left(t\right)\\ x_{\min}\leq x_{K}\left(t\right)\leq x_{\max},\\ y_{\min}\leq y_{K}\left(t\right)\leq y_{\max},\\ h_{\min}\leq h_{K}\left(t\right)\leq h_{\max}, \end{array}$$

where the minimum and maximum values of x, y, and h are grid sizes, indicating the area in which the UAV can fly. We solve this problem using our proposed DQN model and the optimization solver using the Broyden–Fletcher–Goldfarb–Shanno (BFGS) [24] algorithm, and then we compare the results of the two methods.

### 3.2. MDP

Action: a set of actions includes the 3-D movements of the UAV-BS. Accordingly, the UAV-BS has horizontal actions forward (F), backward (B), right (R), left (L), and vertical actions up (U), down (D), and finally staying in place (S). This action set considers a total seven actions, which are expressed as A={F,B,R,L,U,D,S}.

When each action is selected, the UAV-BS moves along the selected direction by a predefined distance $\delta_{m}$. However, when the received average channel power gain of the GUs is lower than a threshold τ, which means the UAV-BS is far from the optimal position, the UAV-BS moves with a larger step size $\Delta_{m}\left(>\delta_{m}\right)$. This mechanism allows the UAV-BS to move to the optimal position quickly when the UAV-BS is initially located far away from the optimal position. In addition, after moving to the optimal position, the staying action prevents the UAV-BS from unnecessary maneuvering.

**Figure 2:**
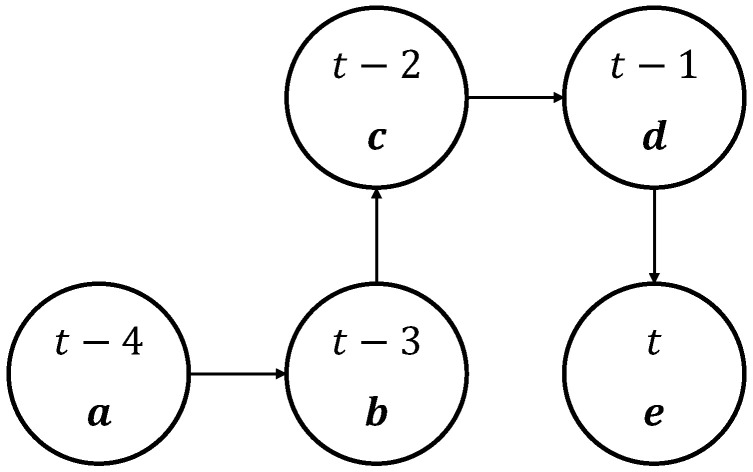
Example of unmanned aerial vehicles serving as base stations (UAV-BS) movement. a, b, c, d, and e mean the average received channel power gains from t−4 to t.

State: from the above action set, three flying directions, i.e., F-B, R-L, and U-D, can be considered. In describing a state in the proposed model, three parameters constitute a state. Specifically, the differences of the average received channel power gain in F-B, R-L, and U-D directions constitute a state vector. For instance, if F action is selected and the UAV-BS moves to a new position, the new average channel power gain is subtracted by the previous value, and F-B direction element of the state vector is updated to this value. The state vector at time t is expressed as $s_{t}\;=\left[\Delta_{FB},\Delta_{RL},\Delta_{UD}\right]$, where $\Delta_{FB},\;\Delta_{RL},$ and $\Delta_{UD}$ are the difference in received channel power gain in the F-B, R-L, and U-D directions, respectively. For instance, let a UAV-BS move in order of R, F, R, and B from t−4 to t and the average received channel power gains be a, b, c, d, and e, as shown in Figure 2. In this case, the state vector at each time step is given in Table 2. 

**Figure 3:**
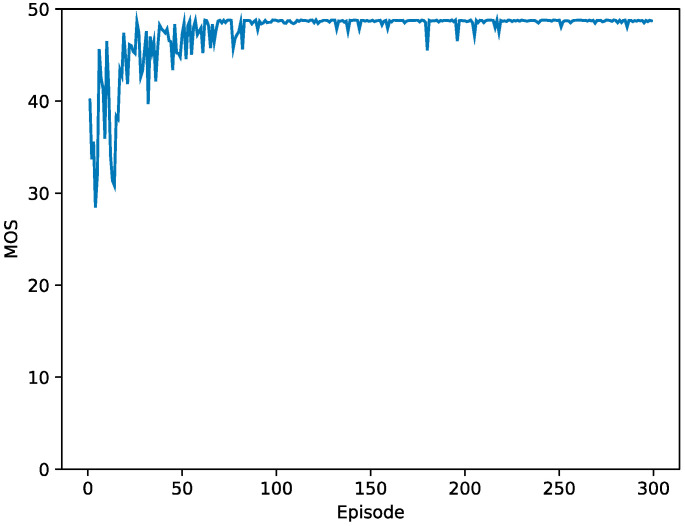
Training process of the deep Q-network (DQN) model.

Reward: The reward at time slot t is expressed as $r_{t}$. The optimal UAV-BS position maximizes the sum of the GUs’ MOS. Accordingly, it is quite a natural and general approach to allocate a positive reward α to an action of increasing MOS and to allocate a negative reward −α to an action of decreasing MOS. Moreover, to prevent the oscillation of the UAV-BS position, a small positive reward β is allocated to an action of retaining current MOS. In the experimental results section, Figure 3 of training process confirms that the reward function operates successfully.

### 3.3. Deep Q-Network (DQN) Algorithm

An ϵ−greedy approach is adopted because exploration for learning is necessary. At the start of the algorithm, the probability of exploration is increased by setting ϵ to 1. Subsequently, the probability ϵ is reduced by multiplying $\epsilon_{\mathrm{decay}}$ at every time step. The UAV-BS moves by $\delta_{m}$ in the selected direction of $a_{t}$. If the randomly chosen action by the ϵ-probability goes out of the area grid, the UAV-BS randomly takes another action. The DQN algorithm for the UAV-BS trajectory model learning is shown in the following Algorithm 1.
**Algorithm 1.** DQN algorithm for UAV-BS trajectory• Initialize the replay memory D• Initialize action-value function Q with random weights• Initialize target-value function Q’ with random weights• Initialize the position of N GUs with R radius.• Set probability ϵ = 1, $\epsilon_{\min}$ = 0.1, $\epsilon_{\mathrm{decay}}$ = 0.999971: **for** episode = 1, ⋯, M do2:          Initialize the position of the UAV-BS3:          **for** t=1, ⋯, T do4:                **if** ϵ> $\epsilon_{\min}$5:                          $\epsilon \;=\;\epsilon \times \epsilon_{\mathrm{decay}}$6:                **end if**7:                Select a random action $a_{t}$ with probability8:                **while** the UAV-BS position goes out of the grid9:                          Select other action except $a_{t}$10:            **end while**11:            otherwise select $a_{t}=\arg\;\max_{a}Q\left(s_{t},a\right)$12:            Execute action $a_{t}$ and observe reward $r_{t}$ and state $S_{t+1}$13:            Store transition $\left(S_{t},a_{t},r_{t},S_{t+1}\right)$ in D14:            Sample random mini-batch of transitions from D15:            Perform a gradient descent to update action-value function Q16:            Every episode update target-value function Q’17:        **end for**18: **end for**

### 3.4. Algorithm Complexity

The complexity of approximating Q function of DQN is affected by the number of states and can be expressed as $O\left({\left|S\right|}^{2}\left|A\right|\right)$ [11]. |S| and |A| represent the numbers of states and actions, respectively. In the proposed model, the number of components in the state vector is fixed to three regardless of the number of the GUs because the average channel power gain is adopted as an input parameter. Therefore, even if the number of GUs increases, the proposed model has an advantage in terms of computational complexity.

## 4. Experimental Results

For the performance analysis, 25 GUs are considered in an area where the grid size is 300 m × 300 m, and the maximum altitude of the UAV-BS is 50 m. The parameter settings for the DQN learning and the experiment parameters are summarized in Table 3 and Table 4, respectively. In determining predefined movement distance $\delta_{m}$, the average UAV-BS altitude 30 m and the average GUs movement per a time slot 1 m are considered, and even when $\delta_{m}$ is reduced to 1 m, MOS gain is not observed, hence, $\delta_{m}=5$ is determined. Initially, the model learns in an environment where the GUs are fixed, and then this learned model is applied to the moving GUs. Note that the model can be trained in both the environments where the GUs are fixed or the GUs move. Since the performance difference is negligible, the model trained with the fixed GUs is preferred in this paper. In the learning stage, 25 GUs are located around the center of an area with 50 m group radius, and it takes 300 episodes with a random UAV-BS initial position in training this model, which has five layers and a rectified linear unit (ReLU) activation function. At the early part of the learning, MOS is about 30, and after the learning, MOS reaches above 50 as shown in Figure 3. In the execution stage, it is assumed that the GUs move between episodes, i.e., no movement within a single episode. At the beginning of the experiment, the position of the UAV-BS is randomly chosen. The position of the UAV-BS and the MOS of GUs are measured by varying the group radius and the path of GUs. In addition, the experimental results are compared with those obtained through the solver of the Python scipy package implemented based on the BFGS algorithm. The solver needs the exact positions of the UAV-BS and all the GUs, and it finds the position of the UAV-BS by locally maximizing the MOS of the GUs. Hence, in order to confirm the optimality of the results of the solver, more than four different initial points are fed to the solver, resulting in the same output of the solver.

**Figure 4:**
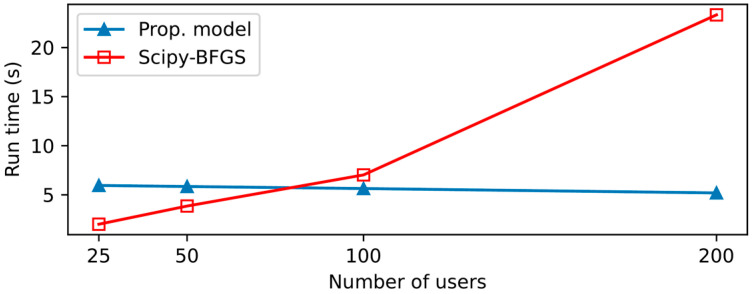
Execution times of two methods with increasing GUs.

Table 5 is a comparison between the proposed algorithm and BFGS. The time complexity of DQN is $O\left({\left|S\right|}^{2}\left|A\right|\right)$, and the algorithm proposed in this paper has a fixed number of inputs (states). Also, this is the time complexity calculated during the training process. When a trained model is applied in UVA-BS network for execution the time complexity is low O(|A|). On the other hand, in the case of BFGS, the time complexity $O\left(n^{2}\right)$ increases with the number of GUs because the location information of all GUs should be received and processed. Moreover, BFGS requires the exact position of each GU as input data, while the proposed model requires the average received channel power gain. The input data requirement for BFGS is quire impractical because it assumes that all the GUs are equipped with GPS and all the GUs’ position are reported to the UAV-BS in a real time. Considering the time complexities of the two methods, the execution time of BFGS is expected to increase sharply as the number of GUs increases, while the proposed algorithm will maintain its execution time even with increasing number of GUs. Moreover, the output of BFGS is the optimal position of the UAV-BS, while the output of the proposed algorithm is the optimal direction to reach the optimal position. Hence, the proposed algorithm requires some iteration before the accumulated optimal directions guide the UAV-BS to the optimal position. This intuition is very well matched with the measured execution time shown in Figure 4. When the number of GUs is small the execution time of BFGS smaller than the proposed algorithm because the proposed algorithm requires some iteration; however, as the number of GUs increases, the execution time of BFGS increases sharply, while the proposed algorithm maintains its execution time regardless of the number of GUs.

**Figure 5:**
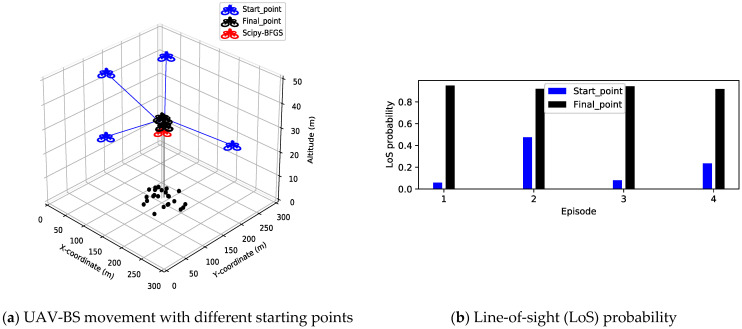
UAV-BS movement and line-of-sight (LoS) probability from random starting positions to the optimal position without considering GU mobility.

**Figure 6:**
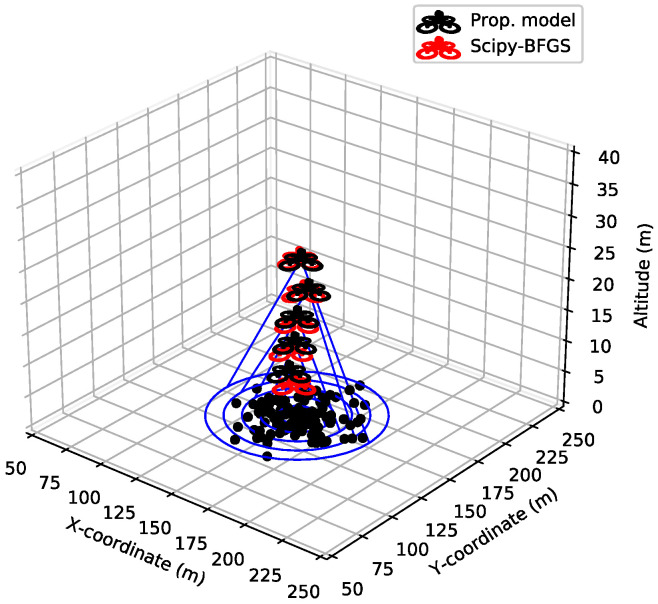
The change of UAV-BS position by varying group radius.

**Figure 7:**
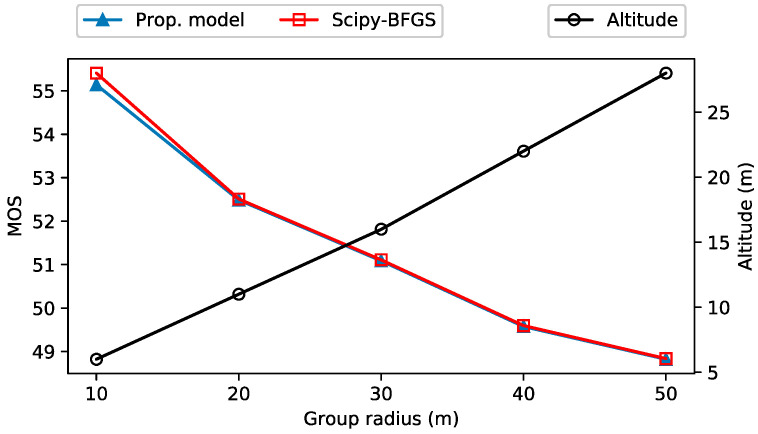
Mean opinion score (MOS) and UAV-BS altitude with varying group radius.

Figure 5, Figure 6 and Figure 7 show the optimal deployment of the UAV-BS and the measured MOS over a single episode. Figure 5 shows the results of an experiment in which group radius is fixed at 50 m. In this figure, even though randomly selected initial points are adopted, the final positions of the learning model result in the nearly same position. Moreover, these final positions have little error compared to the positions obtained by the solver. Moreover, compared with Scipy-BFGS, the MOS gaps between the two methods are negligible. It shows that the proposed model with the predefined moving distance, discrete action setting, and different initial points reaches the same optimal result in terms of both position and performance. Figure 5b shows the LoS probability calculated through Equation (1) at randomly selected initial positions and final positions. When the position of the UAV-BS changes, the elevation angle between the UAV-BS and the GUs changes, so the LoS probability changes. Figure 5b shows the advantage of moving the UAV-BS to the optimal position. As shown in this figure, the probabilities of establishing LoS links with GUs are very low with initial points like 0.05, 0.1, 0.2, 0.5; however, at the final points, these probabilities become higher than 0.9, which results in the improved channel quality between the UAV-BS and the GUs.

Figure 6 and Figure 7 show the optimal UAV-BS altitude and MOS with varying group radius, respectively. The initial group radius is 10 m and increases by 10 m in each episode. As the group radius increases, the NLoS probability for the GUs located near the boundary of group increases. This leads to an increment of the average of NLOS probability. Hence, the UAV-BS starts to decrease the NLoS probability by increasing its altitude. Accordingly, as shown in Figure 6, the altitude of the UAV-BS is linearly proportional to the group radius, approximately 6 m (altitude) per 10 m (radius). However, too high an altitude reduces the MOS of the GUs. Therefore, it is important to find the proper altitude of the UAV-BS.

There are very small discrepancies between the UAV-BS positions out of the proposed model and those of the mathematical solver. Moreover, the difference in terms of MOS is negligible, as shown in Figure 7. This means that the position difference has a very slight effect to the QoE of the GUs. In Figure 7, MOS decreases as the group radius increases. As mentioned above, the wide group radius leads to the high altitude of the UAV-BS, and it results in the decreased received channel power gain and the decreased MOS.

**Figure 8:**
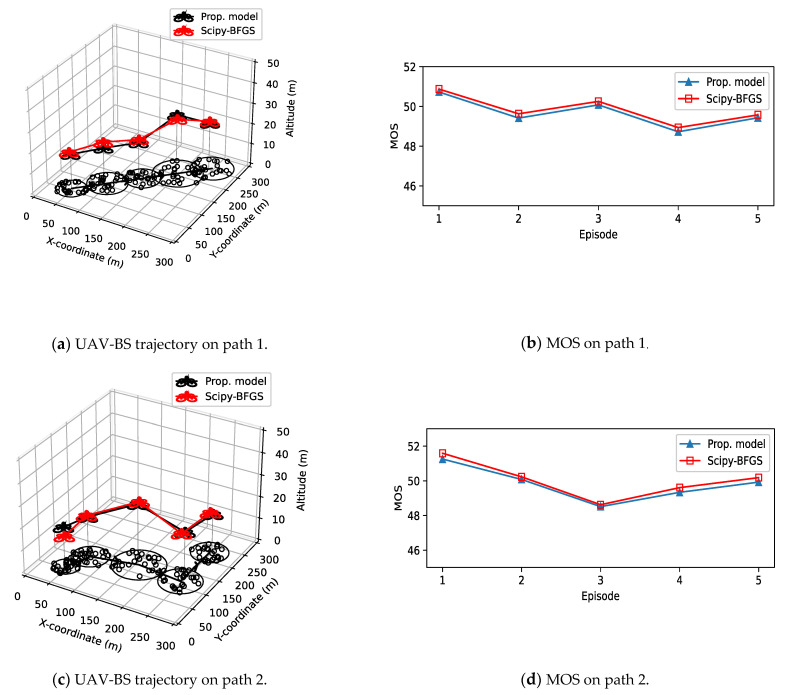

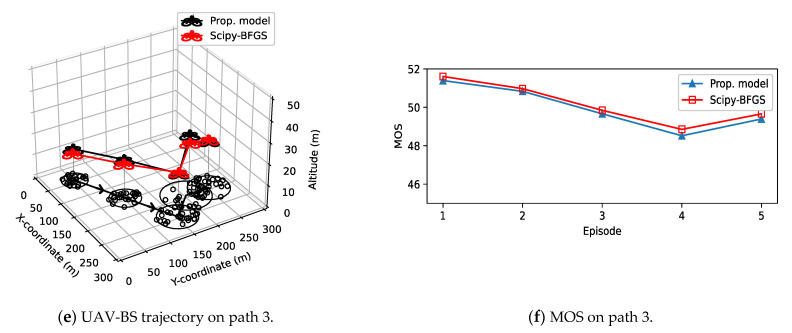
UAV-BS trajectory and MOS with GUs moving in various paths.

The results shown in Figure 8 is very promising and validate the effectiveness of the proposed model. This figure shows the optimal trajectories and the associated MOS curves of the proposed model applied to various paths of the mobile GUs. Note that the model learns in a static environment where the GUs are fixed, then, without the acquisition of the path information and re-learning, this model is applied to the mobile environments where the GUs move randomly around the moving center point. As the GUs move episode by episode, the radius of moving GUs is randomly changed from 25 m to 50 m. In (a) of Figure 8, the GUs move in one direction, and in cases of (c) and (e), the GUs show more dynamic moving patterns. To confirm that the results are not accidental, the experiments are repeated over 50 times, then averaged. In each repetition, the movement of individual GU is randomized with a new random seed. As we can see in Figure 8, even though the situation is so adverse that the GUs’ position information is unavailable and the GUs move with varying group radius, the proposed model successfully locates the UAV-BS at the optimal position. In addition, if the model learns in a dynamic environment where the GUs move randomly episode by episode, the output trajectories are nearly same with those shown in Figure 8. Considering that the proposed DQN model are learned with simplified parameters, and optimal actions selected by the UAV-BS are predefined distance values consisting of seven discrete directions, the proposed algorithm and the optimal position derived by BFGS are very close, as shown in the resulting graph. Moreover, in terms of MOS, the outputs of the proposed model very well matched with those of the solver.

## 5. Conclusions

In this paper, a novel DQN model for an optimal deployment and trajectory design of a UAV-BS is proposed. This model uses only the average channel power gain without accurate location information of GUs. It is confirmed that the proposed model locates UAV-BS where MOS is maximized. Experimental results show that the altitude of UAV-BS increases as the group radius increases. In addition, they demonstrate that 3D trajectory design of UAV-BS is possible using the DQN-model where the model learns in a static environment, and then this model is applied to mobile environments without re-learning.

## Figures and Tables

**Table 1:** Mathematical notations and descriptions.

Notations	Description
$\theta_{i}$	Elevation angle between the unmanned aerial vehicles base station (UAV-BS) and ground user (GU) i
a, b	Environmental parameters
B $,\;B_{i}$	Total Bandwidth/Allocated to GU i
P $,\;p_{i}$	Total transmission power/Allocated to GU i
$\Gamma_{i}\left(t\right)$	Received signal to noise ratio (SNR) of GU i at time slot t
$T_{i}\left(t\right)$	Transmission rate of GU i at time slot t
$N_{0}$	Noise power spectral
$g_{i}\left(t\right)$	Channel gain between the UAV-BS and GU i
α	Path loss exponent
$\mu_{Los},\;\mu_{NLos}$	Attenuation factors for line of sight (LoS) and non-LoS (NLoS)
${\mathrm{MOS}}_{i}\left(t\right)$	MOS of GU i at time slot t
$d\left(T_{i}\left(t\right)\right)$	Delay related to the transmission rate for GU i
TL	Traffic load

**Table 2:** Input vector over time and state.

Time	State $s_{t}=\left[\Delta_{FB},\;\Delta_{RL},\;\Delta_{UD}\right]$
t−4	[0, 0, 0]
t−3	[0, b−a, 0]
t−2	[c−b, b−a, 0]
t−1	[c−b, d−c, 0]
t	[e−d, d−c,0]

**Table 3:** DQN learning parameter settings.

Parameter	Value
Batch size	64
Learning rate	0.001
Size of replay memory	5000
Number of hidden layers	2
Number of neurons in each hidden layer	48
Type of activation function	Rectified linear unit (ReLU)

**Table 4:** Experiment parameter settings.

Time Parameter	Value
Number of users N	25
Group radius R	10–50 m
Carrier frequency f	2 GHz
Transmit power	20 dBm
Bandwidth B	1 MHz
TL	8,000,000 bits
a , b	9.61, 0.16
$C_{1},\;C_{2}$	1.120, 4.6746
Path loss exponent α	2
μLoS	3 dB
μNLoS	23 dB
$\mathrm{Movement}\;\mathrm{distance}\;\delta_{m}\;,\;\Delta_{m}$	5, 10 m
Channel power gain of threshold τ	−100 dBm
α, β	10, 1

**Table 5:** Comparison between the proposed algorithm and Broyden–Fletcher–Goldfarb–Shanno (BFGS).

	BFGS	Proposed Algorithm
**Time complexity**	$\mathrm{High}\;(O\left(n^{2}\right)$)	$\mathrm{Training}:\;\mathrm{High}\;(O\left({\left|S\right|}^{2}\left|A\right|\right))$ Prediction: O(|A|)
**Input**	Exact positions of the UAV-BS and all the GUs	Differences of the average received channel power gain
**Output**	Optimal position(coordinate)	Optimal action(direction)
